# EGFR and SYNE2 are associated with p21 expression and SYNE2 variants predict post-operative clinical outcomes in HBV-related hepatocellular carcinoma

**DOI:** 10.1038/srep31237

**Published:** 2016-08-09

**Authors:** Chuangye Han, Xiwen Liao, Wei Qin, Long Yu, Xiaoguang Liu, Gang Chen, Zhengtao Liu, Sicong Lu, Zhiwei Chen, Hao Su, Guangzhi Zhu, Zili Lu, Zhiming Liu, Xue Qin, Ying Gui, Zengnan Mo, Lequn Li, Tao Peng

**Affiliations:** 1 Department of Hepatobiliary Surgery, the First Affiliated Hospital of Guangxi Medical University, Nanning, 530021, Guangxi Province, China; 2 Department of Pathology, the First Affiliated Hospital of Guangxi Medical University, Nanning, 530021, Guangxi Province, China; 3 Department of General Surgery, the First Affiliated Hospital of Guangxi Medical University, Nanning, 530021, Guangxi Province, China; 4 Department of Clinical Laboratory, the First Affiliated Hospital of Guangxi Medical University, Nanning, 530021, Guangxi Province, China; 5 Department of Clinical laboratory center, the First Affiliated Hospital of Guangxi Medical University, Nanning, 530021, Guangxi Province, China; 6 Center for Genomic and Personalized Medicine, Guangxi Medical University, Nanning, 530021, Guangxi Province, China; 7 Department of Hepatobiliary Surgery, Affiliated Tumor Hospital of Guangxi Medical University, Nanning, 530021, Guangxi Province, China

## Abstract

This study was to explore the association between gene variants and p21 expression and investigate the TP53-independent p21 regulation in hepatitis B virus (HBV) related hepatocellular carcinoma (HCC) patients from Guangxi by genome-wide association study. 426 HBV-related HCC patients were enrolled. Results showed that, after quality control, a total of 21,643 SNPs were identified in 107 p21 positive and 298 p21 negative patients. The variants of epidermal growth factor receptor (EGFR; rs2227983 and rs6950826) and spectrin repeat containing, nuclear envelope 2 (SYNE2; rs8010699, rs4027405 and rs1890908) were associated with p21 expression. Moreover the haplotype block (rs2227983 and rs6950826, r^2^ = 0.378) in EGFR and the haplotype block in SYNE2 (rs8010699 was in strong LD with rs4027405 and rs1890908 (r^2^ = 0.91 and 0.70, respectively)) were identified, and the haplotype A-G of EGFR and haplotype G-A-A of SYNE2 were significantly associated with p21 expression (*P* < 0.01). rs4027405 and rs1890908 were significantly associated with overall survival, and patients with AG/GG genotypes of SYNE2 gene had a worse overall survival (*P* = 0.001, *P* = 0.002). Our findings indicate that variants of EGFR and SYNE2 play an important role in p21 regulation and are associated with the clinical outcome of HBV-related HCC in a TP53-indenpdent manner.

Worldwide, the primary liver cancer has been the 5th most common malignancy in men and the 6^th^ in women^1^. Global Cancer Statistics^1^ in 2012 shows that approximate 782,500 new liver cancer cases and 745,500 deaths occur worldwide in 2012, about half of which are found in China. Moreover, about 70–90% of primary liver cancer is hepatocellular carcinoma (HCC)^2^. There is a geographic difference in the incidence of HCC, which is mainly caused by some etiological variations, with the highest incidence in Eastern, South-Eastern Asia and Northern and Western Africa and the lowest incidence in South-Central Asia and Northern, Central, and Eastern Europe. The major causes of HCC include the infection by hepatitis virus B (HBV) and/or hepatitis C (HCV), aflatoxin-B1 (AFB1) exposure, fasciola hepatica infection and alcoholic cirrhosis^3^,^4^. Both HBV infection and AFB1 exposure are the prominent etiological factors of HCC in Guangxi, a region in southern China^5^.

Hepatocarcinogenesis is an extremely complex process, and there is no a specific molecular mechanism that can elucidate this process. Investigations have demonstrated that the dys-regulation of cell cycle plays an important role in the occurrence and progression of HCC^6^,^7^. p21 is a major cell cycle regulator. It is a cyclin-dependent kinase (CDK) inhibitor and a downstream factor of TP53. It can causes G1 growth arrest and thus it is also as known as a tumor suppressor^8^,^9^. p21 may bind to and inhibit the CDK activity, which mediates its various biological activities and may cause cell cycle arrest at a specific stage^10^,^11^. p21 has been found as one of most important and effective effector molecules of TP53. TP53 is able to bind to the promoter of p21 gene, leading to the direct activation of p21 expression^8^,^12^. Studies have shown that p21 can also be regulated in TP53-independent manners, such as by MYC, E2A and BRCA1^13^,^14^. In addition, it is controversial on whether the HBX gene of HBV affects the p21 regulation. Some studies report that HBX gene can suppress p21 expression^15^,^16^, but this is not found in other studies^17^,^18^.

Although p21 is a tumor suppressor, it may also promote oncogenesis in certain circumstances. p21-deficient mice may spontaneously develop tumors, supporting the tumor suppressor activity of p21^19^. Previous studies have reported that p21 mRNA or protein expression in HCC tissues is often lower than in adjacent normal tissues^20^,^21^,^22^. These results suggest that p21 might act as a tumor suppressor in HCC. Moreover, the spontaneous development of lymphomas is suppressed in p21-deficient mice with TP53- or ATM-deficiency^23^,^24^, indicating that p21 serves as an oncogene. In fact, p21 is over-expressed in a variety of cancers, such as breast cancer, prostate cancer, oesophageal squamous cell carcinoma and cervical cancer, and p21 up-regulation as a poor prognostic biomarker is closely related to the tumor grade, invasiveness and aggressiveness in many cases^11^,^25^,^26^,^27^.

Kao *et al.*^28^ and Zhang *et al.*^29^ reported that p21 expression was a favorable survival factor in HCC, but this was not found in our prior study. This may be ascribed to the difference in patients selected. In their study, the HBV-related and non-HBV-related HCC patients were recruited, but only HBV-related HCC patients were enrolled into our study. Furthermore, different from patients in other regions, patients in Guangxi have a high level exposure to AFB1 in their food which is closely associated with hepatocarcinogenesis^5^. Study has reported that AFB1 exposure leads to a high mutation frequency at *TP53* 249Ser, which has been considered as a molecular fingerprint of HCC pathogenesis^30^. However, at present, whether AFB1 exposure is involved in the regulation of p21 is unclear.

In the present study, a genome-wide association study (GWAS) using Illumina HumanExome BeadChip-12-1_A was performed in Chinese HBV-related HCC patients from Guangxi, aiming to detect the gene variants associated with p21 expression and reveal the TP53-independent p21 regulation.

## Results

### GWAS

#### Baseline Characteristics

The clinicopathological characteristics are shown in Table 1. There were no significant differences in the age, gender, BMI, race, smoking status, drinking status, Barcelona Clinic Liver Cancer (BCLC) stage, serum alpha fetoprotein (AFP) level, transcatheter arterial chemoembolization (TACE) status, pathological grade and *TP53* 249Ser mutation between p21 negative group and p21 positive group (*P* > 0.05). TP53 expression status and hepatic cirrhosis status were significantly different between p21 negative group and p21 positive group (*P* = 0.005 and 0.001, respectively). A significantly positive relationship was observed between p21 expression status and TP53 expression status (r = 0.144, *P* < 0.01).

#### Quality Control

**Figure 1:**
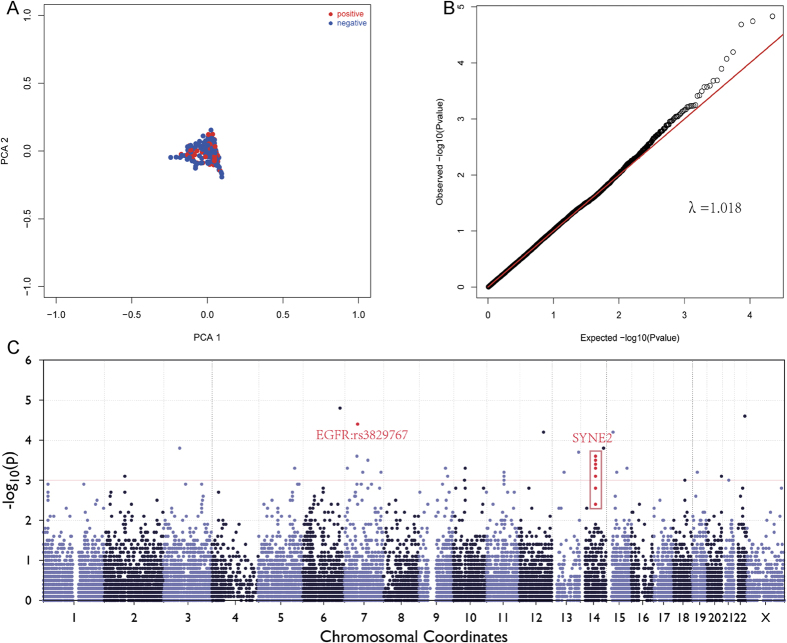
(**A**) Principal components analysis (PCA) for ancestry and population stratification implemented in EIGENSOFT package. The blue dot represents controls and red dot represents patients. No or mild population stratification was found in this study population. (**B**) Quantile-Quantile (Q-Q) plots from Single Variant Test. Reported genomic inflation factor was calculated by MATLAB 7.0, based on P value, and the genomic control inflation factor (λ) was 1.018. (**C**) Manhattan plots for association analysis. Results from Single Variant Test (−log10 P values) were plotted against genomic position (GRCh37/hg19).

After quality control (QC), a total of 21,643 SNPs were identified in 107 p21 positive patients and 298 p21 negative patients. The total genotyping rate in remaining individuals was 98.34%. The principal component analysis (PCA) plot (Fig. 1A) showed that no or mild population stratification in this study population, as indicated by the Quantile-Quantile (Q-Q) plot (Fig. 1B). The genomic control inflation factor (λ) was 1.018.

#### Association Analysis

Due to the limitation of sample size, we focused on the loci with minor allele frequencies (MAF) > 0.05. Manhattan plot is shown in Fig 1C. 30 SNPs in the association analysis are reported in Supplementary Table 2. The SNPs in epidermal growth factor receptor (EGFR) and spectrin repeat containing, nuclear envelope 2 (SYNE2) were associated with p21 expression in HBV-related HCC (Table 2). rs2227983 (MAF = 0.49, *P* = 3.61 × 10^−5^) was located in the exon 13 of EGFR gene and rs6950826 (MAF = 0.32, *P* = 4.06 × 10^−3^) in the intron 7 of EGFR gene. rs8010699 (MAF = 0.19, *P* = 2.41 × 10^−4^), rs3829767 (MAF = 0.20, *P* = 2.94 × 10^−4^), rs4027402 (MAF = 0.22, *P* = 3.60 × 10^−4^), rs9944035 (MAF = 0.10, *P* = 5.46 × 10^−4^), rs4902264 (MAF = 0.19, *P* = 8.03 × 10^−4^), rs4027405 (MAF = 0.16, *P* = 1.71 × 10^−3^) and rs1890908 (MAF = 0.32, *P* = 3.63 × 10^−3^) were found in the SYNE2 gene.

#### Genetic model analysis

Table 3 shows the genetic model considering rs2227983, rs6950826, rs8010699, rs4027405 and rs1890908. The genetic model of rs2227983 was an additive model (AG vs. AA, *P* = 1.26 × 10–3, OR = 3.23, 95%CI = 1.58–6.58; GG vs. AA, *P* = 6.44 × 10–6, OR = 5.65, 95%CI = 2.66–12.00), the G allele was strongly associated with positive p21 expression. The genetic model of rs6950826 was over dominant model (AA/GG vs. AG, *P* = 4.64 × 10^−3^, OR = 2.08, 95%CI = 1.25–3.45). The genetic model of rs8010699 was an additive model (AG vs. GG, *P* = 0.016, OR = 1.83, 95%CI = 1.12–2.99; AA vs. GG, *P* = 3.43 × 10^−3^, OR = 4.12, 95% CI = 1.60–10.64). The genetic model of rs4027405 was a dominant model (AG+GG vs. AA, *P* = 0.018, OR = 1.84, 95% CI = 1.60–10.64). The genetic model of rs1890908 was also a dominant model (AG/AA vs. GG, *P* = 7.8 × 10^−3^, OR = 2.00, 95% CI = 1.20–3.33).

#### Linkage disequilibrium (LD) and Haplotype Analysis

**Figure 2:**
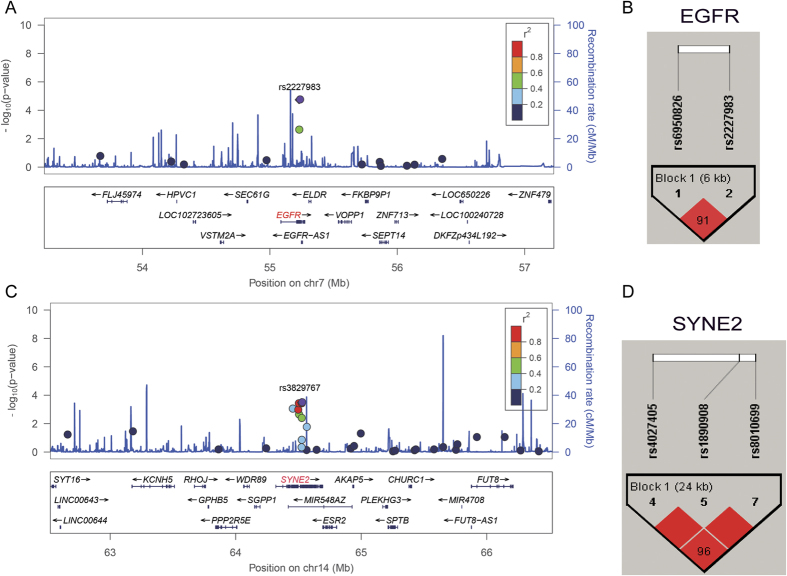
LocusZoom plot for analysis of local linkage disequilibrium (LD) and recombination patterns nearby EGFR (**A**) and SYNE2 (**B**) about 2 Mb. The left vertical axis shows association P-values on the −log10 scale, the right vertical axis shows the recombination rate and the horizontal axis shows the chromosomal position. The bottom of plot shows the near gene. The purple diamond is the most significant SNP at each plot. LD (r2) and recombination rate are estimated from the 1000 Genomes Project ASN data (March2012, build GRCh37/hg19). (**C**) Haploview LD graph of SNPs in EGFR was visualized using the Haploview software 4.2. (**D**) Haploview LD graph of SNPs in SYNE2 was visualized using the Haploview software 4.2. Haploview linkage disequilibrium plots display using the standard color scheme, the number on the cell is the LOD score of D’.

Analysis was performed for LD and haplotype about 2 Mb nearby EGFR and SYNE2 genes (Fig. 2). The haplotype block (rs2227983 and rs6950826, r^2^ = 0.378) in EGFR and the haplotype block in SYNE2 (rs8010699 was in strong LD with rs4027405 and rs1890908 (r^2^ = 0.91 and 0.7, respectively)) were identified, and the haplotype A-G of EGFR and haplotype G-A-A of SYNE2 were significantly associated with p21 expression (*P* = 2.43 × 10^−5^ and 8.63 × 10^−4^, respectively; Table 4).

#### Pathway Analysis and Correlation analysis in mRNA

**Figure 3:**
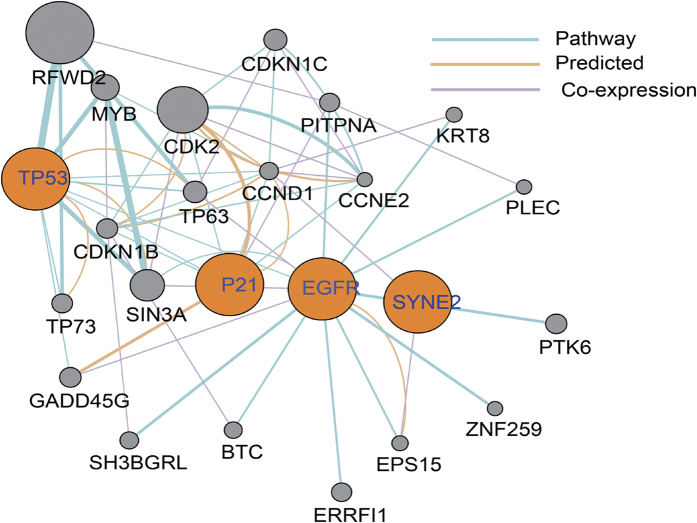
Co-expression/pathway/predicated analysis of p21, SYNE2, EGFR and TP53 according to human expression data in GeneMANIA.

**Figure 4:**
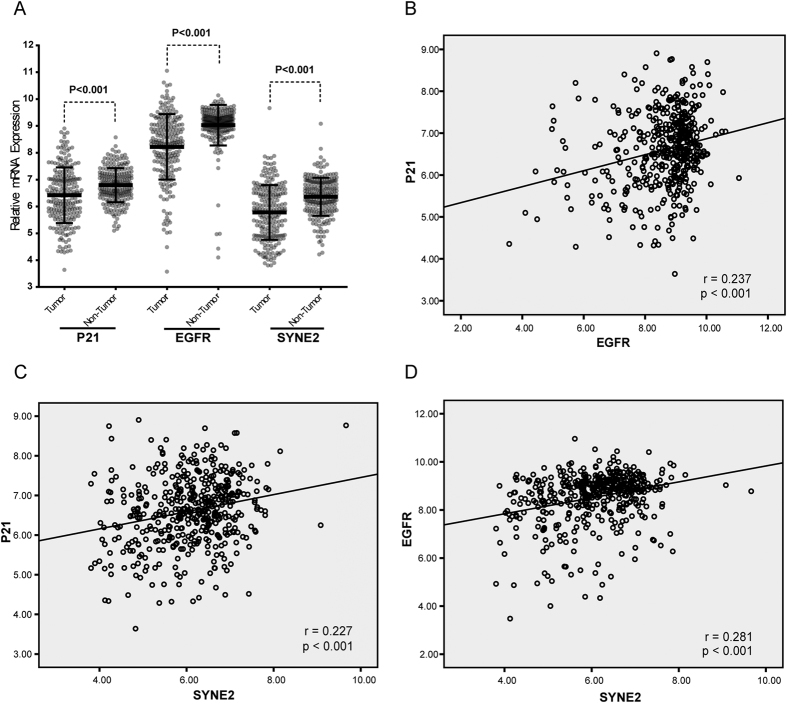
(**A**) Scatter diagram for mRNA expression of p21, SYNE2 and EGFR between HCC tissues and adjacent normal tissues (all *P* < 0.001). (**B**) Correlation analysis for the mRNA expression between p21 and EGFR (*P* < 0.001, r = 0.237). (**C**) Correlation analysis for the mRNA expression between p21 and SYNE2 (*P* < 0.001, r = 0.227). (**D**) Correlation analysis for the mRNA expression between EGFR and SYNE2 (*P* < 0.001, r = 0.281). All data were from Gene Expression Omnibus (GEO accession: GSE14520).

After above analysis, GeneMania Software^31^ was employed (see URLs) to predict the interaction between genes shown in Table 2. Signaling pathway analysis (Fig. 3) showed that p21, SYNE2 and EGFR were in a common pathway or co-expressed. Furthermore, the mRNA expression of p21, SYNE2 and EGFR was compared between HCC tissues and adjacent normal tissues. Data from Gene Expression Omnibus (GEO accession: GSE14520) showed, when compared with adjacent normal tissues, down-regulated expression of p21, SYNE2 and EGFR was observed in HCC tissues (Fig. 4A). Correlation analysis was used to analyze the correlation among p21, SYNE2 and EGFR (Fig. 4B–D). Results showed that there were positive correlations among three genes (SYNE2 vs. p21, *P* < 0.001, r = 0.227; SYNE2 vs. EGFR, *P* < 0.001, r = 0.281; EGFR vs. p21, *P* < 0.001, r = 0.237).

### Survival Analysis

#### Patient’s characteristics and clinical predictors

The clinical and pathologic characteristics of patients are shown in Table 1. Overall, the median follow-up duration was 41 months (range: 3–123 months) and median survival time (MST) was 47 months. In this study, 118 (46.4%) patients died. The 2-year, 5-year and 10-year overall survival (OS) rate was 69.3%, 46.2% and 21%, respectively. As shown Table 1, univariate analysis indicated that patients with BCLC stage B and C (HR = 1.89 and 2.89, respectively), child-pugh class B (HR = 1.54), non-radical resection (HR = 1.37), non-antiviral therapies (HR = 1.6) and portal vein tumor thrombus (PVTT) (PV1–4, HR = 1.85, 2.76, 2.25 and 5.11, respectively) had higher risk for death when compared with patients with BCLC stage A, child-pugh class A, radical resection, antiviral therapies and non-PVTT, respectively. The biobehaviors of HCC, including tumor size (>5 cm), number of tumors (multiple), intrahepatic metastasis and vascular invasion, had adverse effects on the OS.

#### Association of SYNE2 SNPs (rs4027405 and rs1890908) with survival

**Figure 5:**
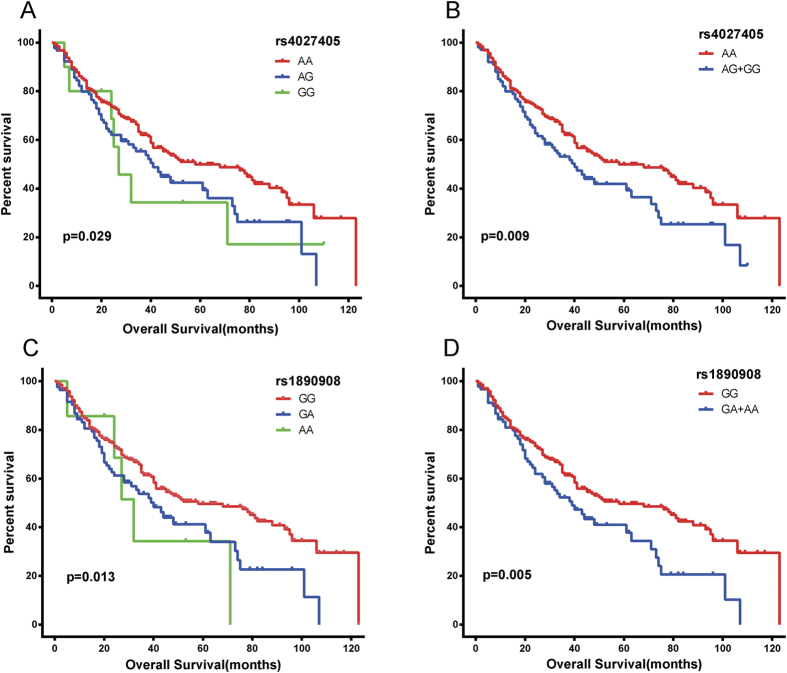
Kaplan-Meir survival curves for SNPs of EGFR and SYNE2. (**A**) rs4027405 (AA vs. AG and GG). (**B**) rs4027405 (AA vs. AG/GG), AG and GG were included into one group. (**C**) rs1890908 (GG vs. AG and AA). (**D**) rs1890908 (GG vs. AG/AA), AG and AA were included into one group.

Cox proportional hazard regression analysis showed rs4027405 and rs1890908 were significantly associated with OS after adjustment for age, body mass index (BMI), smoking status, drinking status, TACE status, serum AFP, radical resection, pathological grade, cirrhosis, intrahepatic metastasis, vascular invasion, antiviral therapies, child-pugh class, BCLC stage and PVTT (Table 5 and Fig. 5). rs4027405 was significantly associated with OS, and patients with AG/GG genotypes had a worse OS (*P* = 0.001, HR = 1.86, 95%CI = 1.28–2.69). Similar findings were also found in rs1890908 (*P* = 0.002, HR = 1.84, 95%CI = 1.26–2.68). In addition, rs2227983, rs6950826 and rs8010699 were not related to the OS.

#### Stratified analysis on the association of SYNE2 SNPs (rs4027405 and rs1890908) with survival

**Figure 6:**
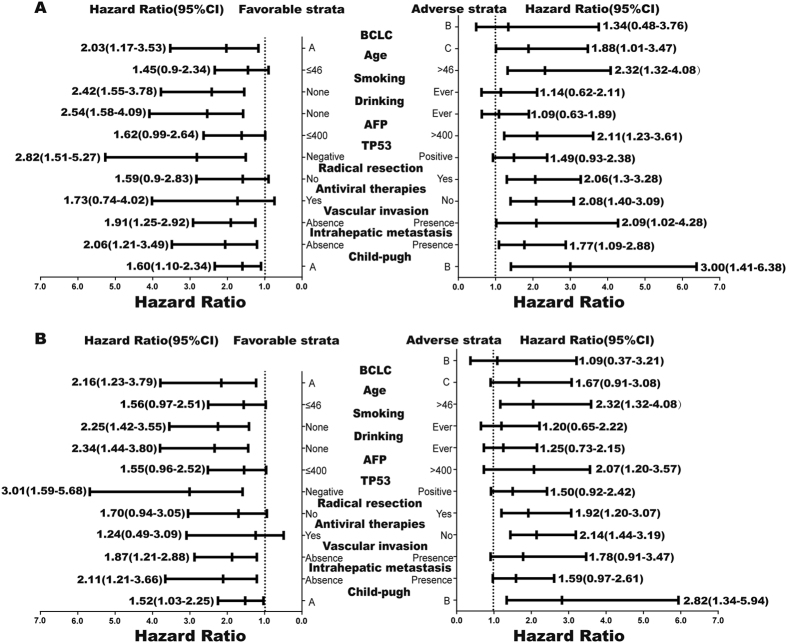
Stratification analysis on the association of rs4027405 (**A**) and rs1890908 (**B**) with clinical outcome of HBV-related HCC patients. HR was indicated for overall survival, stratified by the favorable and adverse outcomes.

A stratified analysis was performed to evaluate the associations of rs4027405 and rs1890908 genotypes with HBV-related HCC survival by age, smoking status, drinking status, serum AFP level, radical resection, TP53 expression status, intrahepatic metastasis, vascular invasion, antiviral therapies, child-pugh class and BCLC stage (Fig. 6). Patients with rs4027405 AG/GG had a poorer OS when they were older than 45 years, or had AFP > 400 ng/ml, non-smoking status, non-drinking status, negative TP53 expression, child-pugh class B or BCLC stage C. Specifically, a significantly increased risk for death conferred by SNP rs4027405 genotypes (AG/GG) was observed in patients negative for TP53 expression (*P* = 0.001, HR = 2.82, 95%CI = 1.51–5.27) or child-pugh class B (*P* = 0.004, HR = 3.00, 95%CI = 1.41–6.38). Similar results were also observed in rs1890908.

#### Combined effect of SYNE2 SNPs (rs4027405 and rs1890908) and serum AFP on OS

The combined effect of SNPs and serum AFP on the OS of HBV-related HCC patients was further analyzed (Table 6). Patients were classified into 4 groups according to the rs4027405 status and serum AFP level: AFP ≤ 400 ng/mL with AA genotype, AFP > 400 ng/mL with AG/GG genotypes, AFP ≤ 400 ng/mL with AA genotype and AFP > 400 ng/mL with AG/GG genotypes. Multivariate Cox regression analysis indicated that, as compared to patients with AA genotype and low serum AFP (AFP ≤ 400 ng/ml), patients with AG/GG genotype or a high serum AFP (AFP > 400 ng/mL) had a significantly higher risk for death (*P* = 0.003, HR = 2.25, 95%CI = 1.31–3.87). Similar results were observed when the rs1890908 and serum AFP level were considered simultaneously (Table 6).

## Discussion

In this study, GWAS was performed to investigate the p21 regulation in Chinese HBV-related HCC patients in Guangxi. Results showed several SNPs in EGFR gene (rs6950826, rs2227983) and SYNE2 gene (rs4027405, rs1890908 and rs8010699) were associated with p21 expression, and the mRNA expression of p21, EGFR and SYNE2 had positive correlations between each other. Furthermore, the p21 expression was significantly associated with the cirrhosis status and TP53 expression, which was consistent with previous results^11^,^32^,^33^, but not related to *TP53* Ser249 mutation (AFB1 exposure).

According to the NCBI database (NG_007726), EGFR gene is mapped to chromosome 7p12 spanning 188.3 kb and contains 30 exons. EGFR is also known as ErbB1 or HER-1 and is a transmembrane receptor belonging to the family of receptor tyrosine kinases (RTK) including ErbB2/HER2, ErbB3/HER3 and ErbB4/HER4^34^. Structurally, these ligands can bind to EGFR system which contains EGF-like domains, including epidermal growth factor (EGF), transforming growth factor α (TGF-α), amphiregulin (AR), epiregulin (EREG), betacellulin (BTC), heparin-binding EGF (HB-EGF) and epigen (EPGN)^35^. The EGFR system is not only essential for the cell proliferation, survival and migration, but closely related to the occurrence, growth and outcome of HCC^36^,^37^,^38^. In addition, in transgenic animals, results showed the over-expression of different EGFR ligands lead to different incidence of HCC, such as TGF-α or EGF over-expression leads to a higher incidence of HCC^39^,^40^,^41^. There is evidence showing that EGFR expression is related to p21 expression^42^, which was confirmed in the present study that there was correlation between EGFR and p21 mRNA expression (Fig. 4). In addition, our results showed rs2227983 (G > A, Arg 521 Lys) and rs6950826 (G > A) of EGFR gene were associated with p21 expression. rs2227983 (G > A, R521K) is located in the exon 13 of EGFR gene. Previous study showed, in metastatic colorectal cancer patients treated with EGFR inhibitor, patients harboring at least one minor allele of rs2227983 G > A were more likely to show a higher tumor response as compared to those with homozygous wild-type allele^43^. In our study, the genetic model of rs2227983 was additive. Patients with allele G had a higher p21 expression. This may be explained as that rs2227983 of EGFR gene may result in an Arg (R) to Lys(K) substitution, leading to the decrease in EGFR activity^44^. Besides, several studies have shown that rs2227983 is also associated with the interstitial lung disease^45^ and gastric cancer^46^. rs6950826 is located in the intron 7 of EGFR gene. To date, no study has been conducted to investigate the association of rs6950826 with the susceptibility to disease or its influence on the clinical outcome of a specific disease. In our study, rs2227983 and rs6950826 were found to be not associated with OS of HBV-related HCC patients. However, how these two loci regulate the expression of EGFR and p21 is still unclear. According to the available findings, we speculate that both loci are likely to affect the function or structure of EGFR gene to regulate the p21 expression, but it is required to be further studied.

In our study, results showed that SNPs (rs4027405, rs1890908 and rs8010699) of SYNE2 gene were associated with p21 expression, and these three loci had strong linkage disequilibrium. Haplotype analysis for the association of SNPs (rs4027405, rs1890908, rs8010699) with p21 expression indicated that patients with haplotype A-G-G had a lower p21 expression in HBV-related HCC. SYNE2, also known as EDMD5, NUANCE and Nesprin-2, is mapped to chromosome 14p23.2 and consists of 116 exons. SYNE2 belongs to the family of giant spectrin-repeat (nesprins), which plays an important role in linking the nucleus to the cytoskeleton and is a key component of the linker of the nucleoskeleton and cytoskeleton (LINC) complex^47^,^48^. Study has reported that SYNE2 depletion may reduce the endothelial cell migration and angiogenic loop formation by regulating the architecture between nucleus and cytoplasm^49^. Recently, nesprins were identified as candidate cancer genes by high-throughput genome sequencing^50^,^51^,^52^. Strikingly, SYNE2 alterations have also been revealed in many cancers, including breast cancer, head and neck cancer and colorectal cancer^50^,^53^,^54^. Meanwhile, our study showed that the mRNA expression of SYNE2 was significantly down-regulated in HCC (Fig. 4A). These findings imply the significant roles of SYNE2 in cancer biology. Currently, how nesprins regulate and control the cancer development and progression is still poorly understood. Derek *et al.*^55^ found that siRNA induced SYNE2 depletion augmented extracellular signal-regulated MAPK1 and 2 (ERK1/2) activity, leading to the increased cell proliferation. p21 has been implicated in cell cycle progression and proliferation via activating ERK1/2 pathway in HCC^56^. Interestingly, the mRNA expression of SYNE2 and p21 is positively correlated in HBV-related HCC (Fig. 4C) and both are involved in the cell cycle progression and proliferation through activating ERK1/2 pathway. On the basis of above findings and our result that SYNE2 variants were associated with p21 expression, we speculate that there is a close relationship between SYNE2 and p21, even SYNE2 is able to regulate p21 expression, but this is needed to be confirmed in future studies.

Kao *et al.*^28^ reported that p21 expression was an independent prognostic factor of favorable survival. Survival analysis in our study showed, although p21 expression was not associated with OS of HBV-related HCC patients, patients positive for p21 expression had higher HR than those negative for p21 expression (HR = 1.13, 95%CI = 0.83–1.54, *P* = 0.443, Table 3). This was not conflicting with above findings. Remarkably, SNPs (rs4027405 and rs1890908) of SYNE2 gene were not only significantly associated with OS, but with p21 expression in HBV-related HCC. The G allele is a risk allele in rs4027405, and G allele carriers have a higher p21 expression and a worse prognosis. Similar results were observed in rs1890908, largely thanking to the strong linkage disequilibrium between it and rs4027405. Sebastian *et al.*^57^ revealed that SYNE2 expression was associated with shorter disease-free survival of gastrointestinal stromal cancer patients. Hence, rs4027405 and rs1890908 are likely to influence the prognosis through affecting the expression or function of SYNE2 and p21. Moreover, univariate and multivariate Cox regression analyses indicated that rs4027405 and rs1890908 were independent predictors of poor OS of HBV-related HCC patients, and it combined with serum AFP can suggest that patients with AFP > 400 ng/ml and higher p21 expression allele have significantly higher risk for death than those with AFP ≤ 400 ng/ml and lower p21 expression allele.

However, our study has several limitations. This was a single center study, and a small fraction of clinical data was missing. The SNPs were not functionally characterized to reveal their effects on the p21 expression and clinical outcome of HBV-related HCC. Therefore, additional studies with larger sample size are needed to further validate the association of these SNPs with p21 expression in HBV-related HCC and with the clinical outcome of HBV-related HCC patients, and *in vitro* studies are required to clarify the underlying mechanisms.

In conclusion, our results demonstrate that SNPs of EGFR (rs6950826 and rs2227983) and SYNE2 (rs4027405, rs1890908 and rs8010699) are associated with p21 expression in HBV-related HCC. rs4027405 and rs1890908 are potential biomarkers for clinical outcome of HBV-related HCC Chinese patients after hepatectomy in Guangxi. In GWAS, TP53 expression as a covariate, and hence, our findings indicate that variants of EGFR and SYNE2 genes may play important roles in the regulation of p21 expression and affect the outcome of HBV-related HCC via TP53-indenpdent manner.

## Methods

### Ethic statement

All experimental protocols were approved by the Ethical Review Committee of the First Affiliated Hospital of Guangxi Medical University (Approval Number: 2015 [KY-E-032]). The methods were carried out in accordance with the approved guidelines. Written informed consent was obtained from all subjects before study.

### Study population

A total of 426 patients with pathologically proven HBV-related HCC were enrolled from the First Affiliated Hospital of Guangxi Medical University between 2005 and 2014. HCC tissues were collected during surgery and immediately stored at −80 °C until the DNA extraction. Total DNA was extracted using the TIANamp Genomic DNA Kit (TIANGEN BIOTECH [BEIJING] CO, LTD). DNA concentration and purity were measured with the NanoDrop2000 system (Thermo Fisher Scientific, Waktham, MA, USA). Immunohistochemical staining of p21 and TP53 in HCC tissues was done in the Department of Pathology at the First Affiliated Hospital of Guangxi Medical University. Finally, 114 patients were found to be positive for p21 expression and 312 negative for p21 expression. Patients with p21 positive expression were included in positive group and those negative for p21 expression of patients in negative group.

According to previous findings^30^, the *TP53* 249Ser mutation was evidence of AFB1 exposure and detected by sanger sequencing after amplification with polymerase chain reaction (PCR) (Sequencing primers in Supplementary Table 1).

The clinicopathological characteristics of included patients were obtained from medical records and pathological reports, including age, gender, smoking status, drinking status, pathological grade, biobehaviors of the cancer, serum AFP level, hepatic cirrhosis, radical resection and use of transcatheter hepatic arterial chemoembolization (TACE). Tumor status was classified according to the Barcelona Clinic Liver Cancer (BCLC) staging system^58^. Child-pugh class was defined as previously published^59^. Portal vein tumor thrombus (PVTT) was determined according to previous classifications^60^: vp1 = PVTT in distal to second order portal branches; vp2 = PVTT in second order portal branches; vp3 = PVTT in first order branches; vp4 = PVTT in main trunk. Smoking status, drinking status and radical resection were defined as previously reported^61^.

### Immunohistochemistry

**Figure 7:**
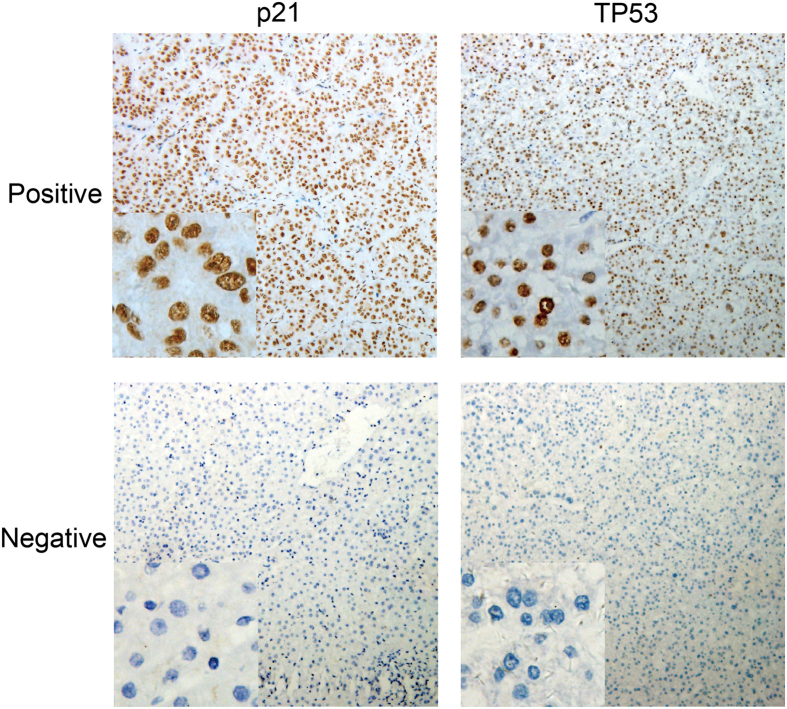
Expression of p21 and TP53 in four hepatocellular carcinoma tissues (Immunohistochemistry). (background: 100×; left down corner: 400×) Fig. 1.

Paraffin embedded HCC tissues were used in immunohistochemistry for p21 and TP53 with general two-step method. Briefly, the antigen retrieval was done with EDTA Tris at a high temperature for 2.5 min. Then, sections were treated with 3% hydrogen peroxide for 10 min to inactivate endogenous peroxidase. After washing thrice with PBS, the mouse against human p21 (1:150) (ZSGB-BIO ORIGENE, Beijing, China) and mouse against human TP53 (1:150) (ZSGB-BIO ORIGENE, Beijing, China) antibodies were independently added, followed by incubation overnight at 4 °C. The sections were incubated with secondary antibody (Dako Cytomation, Glostrup, Denmark) at 37 °C for 30 min. Finally, these sections were visualized with DAB (Dako Cytomation) and counterstained with hematoxylin. Positive and negative controls were included in each immunohistochemical reaction. All the sections were reviewed and scored by two pathologists independently who were blind to the clinical characteristics of patients. At least 10 fields were randomly selected at a high-power (x400 magnifications) at the regions distant from necrotic areas, and the proportion of positive cells was calculated with the following formula: (number of positive cells/total number of the cells ×100%). Positive cells had brown granules in the nucleus. According to previous criteria, positive p21 expression was defined as the presence of ≥5% positive cancer cells^28^,^62^, and positive staining of TP53 as ≥10% positive cancer cells^28^,^63^,^64^ (Fig. 7).

### Genotyping

All samples were genotyped using the Illumina HumanExome BeadChip-12-1_A, which includes 242,901 markers on the protein-altering variants. These markers include non-synonymous single nucleotide polymorphisms (SNPs), SNPs in splice sites, stop variants, SNPs in promoter regions, SNPs in extended MHC region, GWAS tag markers, HLA tags, etc. Details about SNPs can be found at the exome array design webpage. Samples were processed according to the manufacturer’s instructions. Genotype calling was carried out using the Genotyping Module v1.0 in GenomeStudio version 2011.1, and average call rate was 99.84%. A total of 50 samples (>10%) were randomly selected and sequenced for the candidate loci using the ABI Prism 3100 (Applied Biosystems, Shanghai Sangon Biological Engineering Technology & Services Co, Ltd, Shanghai, China). The candidate loci and primers used for sequencing are shown in Supplementary Table 1. The results of sequencing are 100% concordant with the results of genotyping by BeadChip-12-1A.

### Follow up

All patients were followed up via telephone or hospital visit until death or the last time follow-up was done in September 2014. The median follow-up period in 426 patients was 41 months (ranging: 3–123 months), and the median survival time (MST) was 47 months.

### Statistical analysis

#### Quality Control

A quality control (QC) procedure was used before association analysis as follows: 1. The sample was removed if they (i) had an overall genotyping rate of <95%; (ii) had ambiguous gender; (iii) had genome-wide identity-by-descent (IBD) >0.1875; (iv) were outliers in principal components analysis (PCA) for ancestry and population stratification. 2. The SNP was excluded if they had (i) a call rate of <95%; (ii) a P value in Hardy-Weinberg equilibrium (HWE) <1 × 10^−6^; (iii) a Minor allele frequencies (MAF) <0.05. 3. PCA: Guangxi in Southern China is a multi-ethnic region. An analysis of population stratification by PCA^65^ was performed to eliminate the multi-ethnic interference using the EIGENSOFT package. Genomic inflation factors (GIF)^66^ was used to investigate the residual population stratification, which was calculated with MATLAB 7.0 (see URLs). The procedure was performed with the Plink version 1.07, R 3.0.1 and EIGENSOFT package^67^.

#### Association Analysis

The association analysis was evaluated using Single Variant Test^68^ (chose Logistic Score Test) with EPACTS package version 3.2.6 (see URLs), after adjustment for age, gender, BCLC stage, TP53 expression status, TACE status before hepatectomy, pathological grade and hepatic cirrhosis. The Chi-squared test and logistic regression model were independently used to analyze the association of genetic model of SYNE2 and EGFR with p21 expression. Local linkage disequilibrium (LD) and recombination patterns nearby SYNE2 and EGFR were analyzed using the LocusZoom^69^ (see URLs).

#### Survival Analysis

Overall survival (OS) was defined as the time from hepatetomy to death or the last follow-up. MST was calculated for all variables, univariate and multivariate survival analysis were done using the Cox proportional hazard regression model. Hazard ratios (HR) and their 95% confidence interval (CI) were calculated from the Cox proportional hazards regression model with adjustment for age, gender, race, body mass index (BMI), smoking status, drinking status, TACE status, serum AFP level, BCLC stage, child-pugh class, radical resection, pathological grade, hepatic cirrhosis and PVTT. A value of *P* < 0.05 was considered statistically significant. Statistical analysis was performed with SPSS version 18.0 (SPSS, Inc., Chicago, IL, US).

## Additional Information

**How to cite this article**: Han, C. *et al.* EGFR and SYNE2 are associated with p21 expression and SYNE2 variants predict post-operative clinical outcomes in HBV-related hepatocellular carcinoma. *Sci. Rep.* **6**, 31237; doi: 10.1038/srep31237 (2016).

## Supplementary Material

Supplementary Information

## Figures and Tables

**Table 1:** Clinicopathological characteristics of HBV-related HCC patients after data quality control.

Variable	GWAS					Survival Analysis		
	Negative (n = 298)	Positive (n = 107)	OR (95% CI)	P value	Patients (n = 405)	MST (months)	HR* (95% CI)	P* value
Age (years)				0.052				0.708
≤46	154	67	Ref.		221	52	Ref.	
>46	144	40	1.56 (1–2.46)		184	44	1.06 (0.79–1.42)	
Gender				0.619				0.428
Male	268	98	Ref.		366	47	Ref.	
Female	30	9	0.82 (0.38–1.79)		39	82	0.80 (0.46–1.39)	
Race				0.397				0.929
Han	184	71	Ref.		255	47	Ref.	
Minority	114	36	0.82 (0.52–1.30)		150	50	1.01 (0.75–1.38)	
BMI				0.048				0.922
≤25	229	92	Ref.		321	48	Ref.	
>25	69	15	0.54 (0.29–0.99)		84	45	1.02 (0.72–1.45)	
Smoking status				0.506				0.152
None	187	71	Ref.		258	51	Ref.	
Ever	111	36	0.85 (0.54–1.36)		147	39	1.22 (0.92–1.67)	
Drinking status				0.627				0.143
None	173	65	Ref.		238	51	Ref.	
Ever	125	42	0.89 (0.57–1.40)		167	41	1.24 (0.93–1.66)	
Child-pugh class				0.754				0.030
A	238	84			322	50	Ref.	
B	41	16	1.11 (0.59–2.07)		57	31	1.54 (1.04–2.27)	
Missing	19	7			26			
BCLC stage				0.639				<0.001
A	169	59	Ref.		228	95	Ref.	
B	52	16	0.88 (0.47–1.66)		68	39	1.89 (1.27–2.81)	
C	76	32	1.21 (0.73–2.00)		108	27	2.89 (2.09–4.00)	
Missing	1	0			1			
p21 Expression				NA				0.443
Negative	298	0			298	50	Ref.	
Positive	0	107			107	40	1.13 (0.83–1.54)	
TP53 Expression				0.005				0.161
Negative	122	27	Ref.		149	58	Ref.	
Positive	164	74	2.04 (1.24–3.36)		238	41	1.25 (0.92–1.70)	
missing	12	6			18			
AFB1 exposure				0.315				
no	190	74	Ref.		264	50	Ref.	0.364
yes	108	33	0.79 (0.49–1.26)		141	40	1.15 (0.85–1.55)	
TACE status								
before hepatectomy				0.383				0.769
No	235	80	Ref.		315	48	Ref.	
Yes	63	27	1.26 (0.75–2.11)		90	44	1.05 (0.75–1.47)	
after hepatectomy				0.091				0.872
No	134	38	Ref.		172	76	Ref.	
Yes	164	69	1.48 (0.94–2.34)		233	43	1.03 (0.76–1.40)	
Cirrhosis				0.001				0.174
No	22	21	Ref.		43	88	Ref.	
Yes	276	85	0.32 (0.17–0.62)		361	45	1.40 (0.86–2.28)	
missing	0	1			1			
Serum AFP				0.237				0.155
≤400 (ng/ml)	158	48	Ref.		206	51	Ref.	
>400 (ng/ml)	122	49	1.32 (0.83–2.1)		171	41	1.24 (0.92–1.68)	
missing	18	10			28			
Radical resection				0.382				0.034
Yes	163	63	Ref.		226	73	Ref.	
No	127	40	0.82 (0.52–1.29)		167	40	1.37 (1.02–1.84)	
missing	8	4			12			
Pathological grade				0.13				0.618
Well	22	2	Ref.		24	79	Ref.	
Moderately	230	88	4.2 (0.97–18.27)		318	44	1.38 (0.71–2.72)	
Poorly	9	2	2.44 (0.30–20.12)		11	47	1.21 (0.37–3.92)	
missing	37	15			52			
Antiviral therapy				0.001				0.009
Yes	120	23	Ref.		143	>117	Ref.	
No	178	84	2.46 (1.47–4.13)		262	41	1.60 (1.12–2.29)	
Oncological behavior								
Tumor size				0.052				<0.001
≤5 cm	106	27	Ref.		133	123	Ref.	
>5 cm	192	80	1.64 (1–2.69)		272	40	2.08 (1.45–2.96)	
No. of tumors				0.576				0.003
Single (n = 1)	220	76	Ref.		296	58	Ref.	
Multiple (n > 1)	78	31	1.15 (0.70–1.88)		109	34	1.61 (1.17–2.20)	
Regional invasion				0.963				0.129
Absence	254	91	Ref.		345	52	Ref.	
Presence	44	16	1.02 (0.55–1.89)		60	40	1.37 (0.91–2.06)	
Intrahepatic metastasis				0.47				0.001
Absence	155	60	Ref.		216	76	Ref.	
Presence	143	47	1.18 (0.76–1.84)		190	36	1.66 (1.23–2.23)	
Vascular invasion				0.748				<0.001
Absence	241	85	Ref.		326	76	Ref.	
Presence	57	22	1.09 (0.63–1.90)		79	20	2.98 (2.16–4.13)	
PVTT				0.979				
No	250	88	Ref.		338	71	Ref.	<0.001
vp1	8	3	1.07 (0.28–4.10)		11	28	1.85 (0.82–4.23)	
vp2	12	4	0.95 (0.30–3.01)		16	18	2.76 (1.52–5.00)	
vp3	22	10	1.29 (0.59–2.83)		32	22	2.25 (1.44–3.50)	
vp4	6	2	0.95 (0.19–4.78)		8	8	5.11 (2.37–11.02)	

OR = odds ratio, 95% CI = 95% confidence intervals, MST = median survival time, HR = Hazard Ratio, Ref. = Reference.

^*^HR and P value for univariate survival analysis.

**Table 2:** SNPs of EGFR and SYNE2 genes are associated with p21 expression in HBV-related HCC.

SNP	Chr	Position	Gene	Allele	Negative group	Positive group	MAF	P value*
rs2227983	7	55229255	Nonsynonymous: EGFR	G/A	71/135/89	42/52/12	0.49	3.61 × 10^−5^
rs6950826	7	55223176	Intron: EGFR	G/A	158/103/34	34/55/15	0.32	4.06 × 10^−3^
rs8010699	14	64522843	Nonsynonymous: SYNE2	A/G	9/77/204	10/38/55	0.19	2.41 × 10^−4^
rs3829767	14	64519455	Nonsynonymous: SYNE2	A/G	9/80/201	10/38/53	0.20	2.94 × 10^−4^
rs4027402	14	64496749	Nonsynonymous: SYNE2	C/T	12/85/189	15/34/51	0.22	3.60 × 10^−4^
rs9944035	14	64447776	Nonsynonymous: SYNE2	T/C	198/86/8	56/41/9	0.10	5.46 × 10^−4^
rs4902264	14	64491695	Nonsynonymous: SYNE2	T/C	7/82/202	9/37/55	0.19	8.03 × 10^−4^
rs4027405	14	64498037	Nonsynonymous: SYNE2	G/A	4/72/215	8/30/61	0.16	1.72 × 10^−3^
rs1890908	14	64519035	Nonsynonymous: SYNE2	A/G	3/64/227	6/29/68	0.14	3.63 × 10^−3^

^*^Adjustment for age, gender, smoking status, drinking status, BMI, BCLC stage, TP53 expression status, TACE status before hepatectomy, pathological grade and hepatic cirrhosis.

SNP = Single nucleotide polymorphism, OR = odds ratio, 95% CI = 95% confidence intervals. Chr = chromosome; MAF = minor allele frequency.

**Table 3:** Genetic model of rs2227983, rs6950826, rs8010699, rs4027405 and rs1890908.

SNPs	Group		OR* (95% CI)	P value*
	Negative	Positive		
rs2227983 Additive				
AA	89	12	Ref.	3.73 × 10^−5^
AG	135	52	3.23 (1.58–6.58)	1.26 × 10^−3^
GG	71	42	5.65 (2.66–12.00)	6.44 × 10^−6^
rs6950826 Overdominant				
AA/GG	192	49	Ref.	
AG	103	55	2.08 (1.25–3.45)	4.64 × 10^−3^
rs8010699 Additive	129	51		
GG	204	55	Ref.	2.29 × 10^−3^
GA	77	38	1.83 (1.12–2.99)	0.0155
AA	9	10	4.12 (1.60–10.64)	3.43 × 10^−3^
rs4027405 Dominant				
AA	215	61	Ref.	
AG/GG	76	38	1.84 (1.11–3.04)	0.0177
rs1890908 Dominant				
GG	227	68	Ref.	
GA/AA	67	35	2.00 (1.20–3.33)	7.79 × 10^−3^

SNP = single nucleotide polymorphism, OR = odds ratio, 95% CI = 95% confidence intervals. Chr = chromosome; MAF = minor allele frequency, Ref. =reference.

^*^Adjustment for age, gender, smoking status, drinking status, BMI, BCLC stage, TP53 expression status, TACE status before hepatectomy, pathological grade and cirrhosis.

**Table 4:** Associations of haplotypes of EGFR (rs6950826, rs2227983) and SYNE2 (rs4027405, rs1890908 and rs8010699) SNPs with p21 expression.

Haplotypes	Positive (2n)	Negative (2n)	OR (95% CI)	P value
EGFR				
G-A	76	308	Ref.	1.16 × 10^−4^
A-G	88	164	0.46 (0.32–0.86)	2.43 × 10^−5^
Others^a^	50	125	0.62 (0.41–0.93)	0.0219
SYNE2				
A-G-G	153	486	Ref.	3.53 × 10^−3^
G-A-A	45	71	0.49 (0.33–0.75)	8.63 × 10^−4^
Others^b^	16	39	0.76 (0.41–1.40)	0.383

OR = odds ratio, 95% CI = 95% confidence interval, Ref. =reference.

^a^Others include haplotypes G-G and A-A.

^b^Others include haplotypes A-G-A and G-G-G.

**Table 5:** Survival on the basis of genotypes of rs4027405 and rs1890908.

SNP	Patients	MST (months)	Crude HR (95% CI)	P value	Adjusted HR^*^ (95% CI)	P value*
rs4027405						
AA	276	58	Ref.	0.029	Ref.	0.003
AG	102	39	1.48 (1.08–2.04)	0.016	1.81 (1.22–2.67)	0.003
GG	12	27	1.72 (0.84–3.53)	0.141	2.40 (1.05–5.50)	0.039
AG+GG	114	38	1.51 (1.11–2.05)	0.009	1.86 (1.28–2.69)	0.001
Missing	15					
rs1890908						
GG	295	52	Ref.	0.013	Ref.	0.006
GA	93	39	2.10 (0.93–4.78)	0.076	2.04 (0.84–4.92)	0.115
AA	9	27	1.52 (1.10–2.11)	0.011	1.81 (1.22–2.70)	0.003
GA+AA	102	38	1.57 (1.15–2.14)	0.005	1.84 (1.26–2.68)	0.002
Missing	8					

HR = hazard ratio, 95% CI = 95% confidence interval, MST = median survival time, Ref. =reference.

^*^Adjustment for age, gender, BMI, race, smoking status, drinking status, child-pugh class, cirrhosis, BCLC stage, pathological grade, TACE status post hepatectomy, antiviral therapy after hepatectomy, radical resection, intrahepatic metastasis, vascular invasion and PVTT.

**Table 6:** Combined effects of SNPs (rs4027405, rs1890908) and serum AFP level.

SNP	MST (months)	Crude HR (95% CI)	P	Adjusted HR* (95% CI)	P*
rs4027405					
AFP ≤ 400+AA	68	Ref.	0.005	Ref.	0.012
AFP ≤ 400+AG/GG	52	1.27 (0.87–1.87)	0.220	1.09 (0.69–1.72)	0.703
AFP > 400+AA	39	1.63 (1.05–2.52)	0.029	1.63 (1.00–2.65)	0.048
AFP > 400+AG/GG	28	2.25 (1.40–3.61)	0.001	2.25 (1.31–3.87)	0.003
rs1890908					
AFP ≤ 400+GG	68	Ref.	0.006	Ref.	0.018
AFP ≤ 400+AG/AA	52	1.24 (0.86–1.79)	0.254	1.09 (0.71–1.69)	0.683
AFP > 400+GG	39	1.62 (1.04–2.52)	0.031	1.63 (1.00–2.66)	0.050
AFP > 400+AG/AA	28	2.23 (1.40–3.78)	0.001	2.21 (1.26–3.88)	0.006

HR = hazard ratio, 95% CI = 95% confidence interval, MST = median survival time, Ref. = reference.

^*^Adjustment for age, gender, BMI, race, smoking status, drinking status, child-pugh class, cirrhosis, BCLC stage, pathological grade, TACE status post hepatectomy, antiviral therapy after hepatectomy, radical resection, intrahepatic metastasis, vascular invasion and PVTT.
