# Supplementary material for: EGFR and SYNE2 are associated with p21 expression and SYNE2 variants predict post-operative clinical outcomes in HBV-related hepatocellular carcinoma
Source: Sci Rep. 2016 Aug 9;6:31237. doi: 10.1038/srep31237 (PMC4977508; doi:10.1038/srep31237)
Supplement: Supplementary Information [file srep31237-s1.doc]

**EGFR and SYNE2 are associated with p21 expression and SYNE2 variants** **predict** **post-operative clinical outcomes in** **HBV-related hepatocellular carcinoma**

Chuangye Han^1*^, Xiwen Liao^1*^, Wei Qin^1*^, Long Yu^1^, Xiaoguang Liu^1^, Gang Chen^2^, Zhengtao Liu^1^, Sicong Lu^1^, Zhiwei Chen^1^, Hao Su^1^, Guangzhi Zhu^1^, Zili Lu^2^, Zhiming Liu^3^, Xue Qin^4^, Ying Gui^5^, Zengnan Mo^6^, Lequn Li^7^, Tao Peng^1^

*^1^Department of Hepatobiliary Surgery, ^2^Department of Pathology, ^3^Department of General Surgery, ^4^Department of Clinical Laboratory, ^5^Department of Clinical laboratory center, the First Affiliated Hospital of Guangxi Medical University, Nanning, 530021, Guangxi Province, China; ^6^Center for Genomic and Personalized Medicine, Guangxi Medical University, Nanning, 530021, Guangxi Province, China; ^7^Department of Hepatobiliary Surgery, Affiliated Tumor Hospital of Guangxi Medical University, Nanning, 530021, Guangxi Province, China*

**These authors contribute to this article equally*

Supplementary table 1. Primers for polymerase chain reaction

| SNP | Primers | Sequences(5＇—— 3＇) | Annealing temperature(℃) | Amp Length(bp) |
| --- | --- | --- | --- | --- |
| rs2227983 | Forward | CTGACTGCTGTGACCCACTC | 62 | 226 |
|  | Reverse | TAACAACAACCTGGAGCCTTATTTTTGA |  |  |
| rs6950826 | Forward | CCAAGAGCAGATGGTTCACAGA | 60 | 607 |
|  | Reverse | AGAAGGAATCCCGTTCAAGAGT |  |  |
| rs8010699 | Forward | GCATCACTTCCCTCGAAGCCA | 62 | 598 |
|  | Reverse | GGAGGAGCACACAAGAGAACTCG |  |  |
| rs3829767 | Forward | AATTGACAGTGGAATCTCAACACATCT | 62 | 258 |
|  | Reverse | TCTTGATTAAGTAATAGTTCATTGCGCTGTA |  |  |
| rs4027402 | Forward | TGTGAGTGAAAACAATCAGAGAGAAATAATTTG | 62 | 254 |
|  | Reverse | TGTCTATCATGTCTGGACACCGT |  |  |
| rs9944035 | Forward | TCAGAGTACATATTATTTTGCCGCTGT | 62 | 260 |
|  | Reverse | GCCCTTAGTAAGCGAAGGTTTTC |  |  |
| rs4902264 | Forward | CAAGGTTTGTGCCATCCTTTTCTTT | 63 | 252 |
|  | Reverse | CATGGAAGAAACCATAATCTCAGGTAATGTAT |  |  |
| rs4027405 | Forward | CACTTCAGTGTAAACAAAAAGATTTGGAAAAC | 62 | 250 |
|  | Reverse | GTCTTGAAAGTGCAATCACAAGCA |  |  |
| rs1890908 | Forward | CACATGATGGCATTCTAGCTAGGC | 62 | 248 |
|  | Reverse | ACTTTCTTGAACCATAAGTTGAAACTTCCTTA |  |  |
| TP53 249Ser* | Forward | CTTGCCACAGGTCTCCCCAA | 61 | 237 |
|  | Reverse | AGGGGTCAGCGGCAAGCAGA |  |  |

SNP = single nucleotide polymorphisms.

*TP53 249Ser mutation

Supplementary table 2. Results from Single Variant Tests

| SNP | Chr | Position | Gene | Minor/major allele | Positive  group | Negative  group | MAF | P value* |
| --- | --- | --- | --- | --- | --- | --- | --- | --- |
| rs1471218 | 6 | 153863384 | Intergenic | G/A | 7/30/69 | 45/127/117 | 0.33 | 1.45×10^-5^ |
| rs148258530 | 22 | 46654664 | Nonsynonymous:PKDREJ | G/T | 85/18/4 | 277/20/1 | 0.06 | 2.65×10^-5^ |
| rs2227983 | 7 | 55229255 | Nonsynonymous:EGFR | G/A | 42/52/12 | 71/135/89 | 0.49 | 3.61×10^-5^ |
| rs10777944 | 12 | 99158757 | Intron:ANKS1B | C/A | 25/59/22 | 43/128/120 | 0.41 | 6.63×10^-5^ |
| rs1432133 | 15 | 27228345 | Intron:GABRG3 | C/T | 77/27/3 | 258/36/1 | 0.09 | 6.64×10^-5^ |
| rs11705970 | 3 | 66714410 | Intergenic | C/T | 64/34/7 | 118/132/44 | 0.34 | 1.44×10^-4^ |
| rs17093294 | 14 | 96178688 | Nonsynonymous:TCL1A | C/T | 97/9/0 | 218/71/4 | 0.11 | 1.68×10^-4^ |
| rs12871532 | 13 | 108668547 | Intergenic | T/C | 23/36/44 | 20/107/157 | 0.30 | 2.24×10^-4^ |
| rs8010699 | 14 | 64522843 | Nonsynonymous:SYNE2 | A/G | 10/38/55 | 9/77/204 | 0.19 | 2.41×10^-4^ |
| rs2240089 | 7 | 51096036 | Nonsynonymous:COBL | G/C | 56/44/7 | 99/160/39 | 0.37 | 2.73×10^-4^ |
| rs2240089 | 7 | 97816327 | Intron:LMTK2 | C/T | 69/32/6 | 232/63/1 | 0.14 | 2.94×10^-4^ |
| rs3829767 | 14 | 64519455 | Nonsynonymous:SYNE2 | A/G | 10/38/53 | 9/80/201 | 0.20 | 2.94×10^-4^ |
| rs4027402 | 14 | 64496749 | Nonsynonymous:SYNE2 | C/T | 15/34/51 | 12/85/189 | 0.22 | 3.60×10^-4^ |
| rs3173615 | 7 | 12269417 | Nonsynonymous:TMEM106B | C/G | 21/49/35 | 28/119/144 | 0.34 | 4.48×10^-4^ |
| rs7097397 | 10 | 50025396 | Nonsynonymous:WDFY4 | G/A | 17/43/46 | 16/107/168 | 0.27 | 4.80×10^-4^ |
| rs34787999 | 9 | 108366734 | Nonsynonymous:FKTN | G/A | 82/21/3 | 268/29/1 | 0.07 | 5.03×10^-4^ |
| rs2562784 | 15 | 84286492 | Intron:SH3GL3 | A/G | 32/52/20 | 143/116/32 | 0.34 | 5.18×10^-4^ |
| rs9944035 | 14 | 64447776 | Nonsynonymous:SYNE2 | T/C | 56/41/9 | 198/86/8 | 0.10 | 5.46×10^-4^ |
| rs3828609 | 5 | 149432863 | Utr3:CSF1R | T/C | 84/23/0 | 271/25/1 | 0.06 | 5.61×10^-4^ |
| rs13225097 | 7 | 150307167 | Intergenic | A/G | 80/20/1 | 180/97/14 | 0.18 | 5.82×10^-4^ |
| rs7998781 | 13 | 49138910 | Intergenic | C/T | 76/23/7 | 238/43/3 | 0.11 | 6.45×10^-4^ |
| rs660339 | 11 | 73689104 | Nonsynonymous:UCP2 | G/A | 27/56/22 | 127/124/39 | 0.38 | 6.53×10^-4^ |
| rs740158 | 7 | 77055836 | Intergenic | T/C | 69/32/6 | 230/66/1 | 0.14 | 6.56×10^-4^ |
| rs8024461 | 15 | 44582886 | Intron:CASC4 | G/A | 8/36/60 | 39/139/114 | 0.34 | 6.58×10^-4^ |
| rs1781873 | 19 | 21477431 | Nonsynonymous:ZNF708 | T/C | 12/34/56 | 57/125/103 | 0.38 | 6.99×10^-4^ |
| rs117284777 | 2 | 85625892 | Nonsynonymous:CAPG | T/C | 88/17/2 | 274/23/0 | 0.05 | 7.14×10^-4^ |
| rs3810485 | 20 | 62194128 | Nonsynonymous:RP4-697K14 | G/A | 44/53/10 | 173/113/11 | 0.26 | 7.96×10^-4^ |
| rs2444601 | 11 | 73611016 | Intron:PAAF1 | T/C | 30/52/24 | 131/119/41 | 0.38 | 8.00×10^-4^ |
| rs4902264 | 14 | 64491695 | Nonsynonymous:SYNE2 | T/C | 9/37/55 | 7/82/202 | 0.19 | 8.03×10^-4^ |

* Adjustment for age, gender, smoking status, drinking status, BMI, BCLC stage, TP53 expression status, TACE status before hepatectomy, pathological grade and hepatic cirrhosis.

SNP = single nucleotide polymorphism. Chr = chromosome; MAF = minor allele frequency.

Top 30 genes listed by the order of related P value
